# Hypothesis that ancestral eukaryotes sexually proliferated without kinetochores or mitosis

**DOI:** 10.1242/jcs.263843

**Published:** 2025-06-10

**Authors:** Bungo Akiyoshi

**Affiliations:** Institute of Cell Biology, School of Biological Sciences, University of Edinburgh, Max Born Crescent, Edinburgh EH9 3BF, UK

**Keywords:** Meiosis, Mitosis, Eukaryote, Prokaryote, Eukaryogenesis, Kinetochore, Genetic exchange, Cell fusion

## Abstract

Eukaryotes possess two different mechanisms to transmit genetic material – mitosis and meiosis. Because mitosis is universal in all present-day eukaryotes, it has been widely assumed, despite the absence of definitive evidence, that meiosis evolved from mitosis during eukaryogenesis. In both processes, chromosome movement depends on interactions between spindle microtubules and a macromolecular protein complex called the kinetochore that assembles onto centromere DNA. Spindle microtubules consist of α- and β-tubulin subunits, which are conserved in all studied eukaryotes. Similarly, canonical kinetochore components are found in almost all eukaryotes. However, an evolutionarily divergent group of organisms called kinetoplastids has a unique set of kinetochore proteins. It remains unclear why and when different types of kinetochores evolved. In this Hypothesis article, I propose that the last eukaryotic common ancestor (LECA) did not have a kinetochore and that these two kinetochore systems evolved independently – one in the ancestor of kinetoplastids and another in the ancestor of all other eukaryotes. Based on the notion that archaea and the LECA possessed cell fusion and genetic exchange machineries, I further propose that key aspects of meiosis evolved prior to mitosis, challenging the dogma that meiosis evolved from mitosis.

## Teaser

**Summary:** This Hypothesis article proposes that key aspects of meiosis evolved prior to mitosis in ancestral eukaryotes.

## Introduction

All living organisms on earth belong to the bacteria, archaea or eukaryotes. Accurate transmission of genetic material is essential for the survival of all domains of life. The genomes of prokaryotes (bacteria and archaea) typically exist in the form of a circular chromosome located in the cytoplasm. In bacteria, the spontaneous entropy-driven de-mixing of chromosomes that occurs as DNA is replicated within a confined space (i.e. the cell membrane) is thought to be a major mechanism of genome segregation (Jun and Mulder, 2006; Jun and Wright, 2010) (Box 1). As the genomes and cells of archaea are similar in size, shape and structure to those of bacteria (Toro and Shapiro, 2010; Badrinarayanan et al., 2015; Barillà, 2016), this mechanism could also in principle apply to archaea. Compared to prokaryotes, eukaryotic genomes are more complex, typically existing in the form of multiple linear chromosomes contained in a membrane-bound structure called the nucleus. Chromosome segregation in eukaryotes differs significantly from that in prokaryotes and requires an active mechanism. Eukaryotes are thought to have evolved from an archaeal host that incorporated a bacterial symbiont as an efficient energy source, and the formation of the nucleus was likely a key event during eukaryogenesis (Embley and Martin, 2006; Martin and Koonin, 2006; Koonin, 2015; Spang et al., 2015; Baum and Spang, 2023; Vosseberg et al., 2024). Despite the essentiality of genetic inheritance for the survival of all organisms, the mechanism of chromosome segregation used during the transition from prokaryotes to eukaryotes has rarely been discussed.

Box 1. How do prokaryotes segregate their genome? Despite several decades of research, knowledge of the mechanism of prokaryotic chromosome segregation remains elusive (Reyes-Lamothe et al., 2012; Gogou et al., 2021). Although the ParAB system found in many bacteria plays an essential role in plasmid segregation (Gerdes et al., 2010; Jalal and Le, 2020; Pulianmackal and Vecchiarelli, 2024), it is often dispensable in genome segregation. Deletion mutants of the ParA ATPase can be created without significant growth perturbation in many bacteria, including *Bacillus subtilis* (Ireton et al., 1994), *Pseudomonas putida* (Godfrin-Estevenon et al., 2002) and *Streptomyces coelicolor* (Kim et al., 2000). The most conserved player known to be involved in bacterial chromosome segregation is the SMC complex, which compacts DNA to facilitate segregation (Goloborodko et al., 2016; Mäkelä and Sherratt, 2020; Harju et al., 2024). However, even the SMC complex can be mutated or deleted in *E. coli* (Niki et al., 1991), *Streptococcus pneumoniaemmi* (Minnen et al., 2011), *Mycobacterium tuberculosis* (Güthlein et al., 2008), *Staphylococcus aureus* (Yu et al., 2010) and *Deinococcus radiodurans* (Bouthier de la Tour et al., 2009).

So far, very little is known about the mechanism of chromosome organization and segregation in archaea (Herrmann and Soppa, 2002; Long and Faguy, 2004; Ettema et al., 2011; Barillà, 2016; Kamada and Barillà, 2018; Takemata et al., 2019; Takemata and Bell, 2020; Cezanne et al., 2024; Wollweber et al., 2025). Like bacteria, many archaea have a SegAB system, composed of SegA (an ortholog of ParA ATPase) and SegB (a site-specific DNA-binding protein) (Kalliomaa-Sanford et al., 2012; Yen et al., 2021). However, deletion mutants of SegAB can be isolated in *Saccharolobus acidocaldarius* and *Sulfolobus acidocaldarius*, meaning that SegAB is not essential for their proliferation (Charles-Orszag et al., 2025 preprint; Kabli et al., 2025 preprint). Essentially nothing is known about the mechanism of chromosome segregation in Asgard (now called Promethearchaeota), the closest prokaryotic relative of eukaryotes known to date. It is noteworthy that all the Asgard strains isolated so far grow extremely slowly (doubling time >7 days) (Imachi et al., 2020, 2025 preprint; Rodrigues-Oliveira et al., 2023). To gain insights into the evolution of chromosome segregation mechanisms during eukaryogenesis, it will be important to understand the mode of genome segregation used by Asgard archaea.

**Fig. 1.**
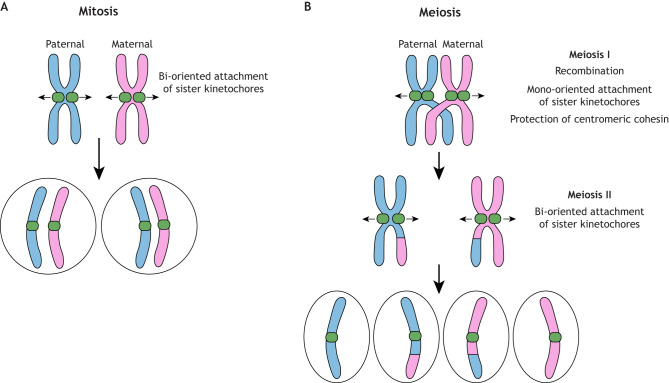
**Mitosis and meiosis in present-day eukaryotes.** Mitosis (left). Sister kinetochores on duplicated chromosomes form bi-oriented attachments to spindle microtubules, which segregate sister chromatids away from each other. Meiosis (right). Paternal and maternal chromosomes undergo recombination, creating linkages between homologous chromosomes. During meiosis I, sister kinetochores attach to spindle microtubules emanating from the same pole (called mono-orientation) so that homologous chromosomes are segregated away from each other. Centromeric cohesin must be protected from degradation so that sister kinetochores can form bi-oriented attachments during meiosis II. Mitosis is a clonal amplification process, whereas meiosis produces four haploid cells with unique genetic contents.

In present-day eukaryotes, genome duplication and transmission occur in a defined cell division cycle (Novak et al., 1998; Morgan, 2007). Chromosome replication occurs during S phase and duplicated sister chromatids are held together by the cohesin complex until they are separated in M phase (Yatskevich et al., 2019). Chromosome segregation is driven by spindle microtubules, dynamic polymers consisting of tubulin subunits, which move chromosomes by interacting with the kinetochore, a macromolecular protein complex that assembles onto centromere DNA (Mitchison and Kirschner, 1984; McIntosh, 2016; Musacchio and Desai, 2017). There are two types of cell division – mitosis and meiosis. Mitosis is a clonal amplification process in which one round of DNA replication is followed by one round of chromosome segregation, producing two genetically identical daughter cells (Fig. 1). By contrast, in meiosis, one round of DNA replication (typically in a diploid cell) is followed by two rounds of chromosome segregation – meiosis I, in which homologous chromosomes are separated, followed by meiosis II, in which sister chromatids are separated, producing four haploid cells each with unique genetic contents (Fig. 1) (Petronczki et al., 2003; Loidl, 2016). A key aspect of meiosis is genetic exchange between paternal and maternal chromosomes, which creates genetic diversity among the population (Zickler and Kleckner, 2023). At the beginning of meiosis, sister chromatids held together by cohesin, as in mitosis, are organized into a linear array of loops emanating from a structure called the axis, which contains cohesin and axial element proteins (Ur and Corbett, 2021). Meiotic recombination is initiated by programmed double-strand DNA breaks induced by the topoisomerase-like protein Spo11 (Arter and Keeney, 2024; Chen and Weir, 2024). Some double-strand breaks turn into crossovers by maturing into recombination intermediates, called chiasmata, that physically link homologous chromosomes together (Hunter, 2015; Pyatnitskaya et al., 2019). The synaptonemal complex, a meiosis-specific structure, forms between homologous chromosomes during the prophase of meiosis I to promote genetic exchange (Adams and Davies, 2023). Another unique feature of meiosis is mono-orientation of sister kinetochores, which allows segregation of homologous chromosomes rather than sister chromatids, as well as protection of centromeric cohesin during meiosis I, which allows segregation of sister chromatids during meiosis II (Nasmyth, 2015; Koch and Marston, 2025) (Fig. 1). Except for recombination proteins and topoisomerases, many of the factors involved in eukaryotic chromosome segregation are not present in prokaryotes (e.g. kinetochores, cohesin and synaptonemal complexes). Therefore, understanding when and how these eukaryote-specific features evolved could provide important insights into the mechanism of eukaryogenesis.

In this Hypothesis article, I first introduce the current dogma and then present an alternative view with regard to the origin of meiosis. By speculating on the possible evolutionary origins of two distinct kinetochore systems, I then propose that ancestral eukaryotes proliferated via a primitive meiosis-like mechanism prior to the invention of a kinetochore-dependent genome segregation system. Finally, several possible implications of this alternative view are also discussed.

## The dogma – meiosis evolved from mitosis

Given that mitosis is universal in all eukaryotes, it is generally assumed that mitosis was present in the last eukaryotic common ancestor (LECA) from which all present-day eukaryotes originated. Similarly, because many genes involved in meiosis are present in diverse eukaryotic organisms, it is thought that meiosis was also present in the LECA, with loss in some lineages (Ramesh et al., 2005; Speijer et al., 2015; Bloomfield, 2016; Colnaghi et al., 2022). In contrast, prokaryotes have neither mitosis nor meiosis, meaning that these mechanisms evolved at some point during eukaryogenesis. In my view, no strong evidence is available to determine the temporal order of the evolution of these two related processes. Nonetheless, because mitosis is universal in all eukaryotes, it has been assumed by many researchers that mitosis evolved first and that meiosis subsequently evolved from mitosis (Cleveland, 1947; Maynard Smith, 1978; Hurst and Nurse, 1991; Maguire, 1992; Kleckner, 1996; Cavalier-Smith, 2002, 2010b; Solari, 2002; Wilkins and Holliday, 2009; Goodenough and Heitman, 2014; Lenormand et al., 2016).

## An alternative view – primitive meiosis evolved prior to mitosis

Garg and Martin challenged this assumption by arguing that meiosis/sex must have evolved first (Garg and Martin, 2016), based on the idea that for long-term survival, all species must use recombination to avoid accumulation of deleterious mutations (the so-called Muller's rachet) (Muller, 1932, 1964; Felsenstein, 1974; Kondrashov, 1994). Prokaryotes can bring in external DNA for recombination using lateral gene transfer mechanisms, such as transformation and conjugation (Chen et al., 2005). Based on the idea that prokaryotic lateral gene transfer mechanisms were largely lost at eukaryotic origin (Ku et al., 2015; Martin, 2018), Garg and Martin argued that meiosis/sex must have evolved prior to mitosis in order to avoid Muller's rachet during the prokaryote-to-eukaryote transition. In their paper (Garg and Martin, 2016), they stated that “eukaryotic chromosome division arose in a filamentous, syncytial, multinucleated ancestor, in which nuclei with insufficient chromosome numbers could complement each other through mRNA in the cytosol and generate new chromosome combinations through karyogamy”. To my knowledge, this is the first paper that challenged the dogma that meiosis evolved from mitosis, which was further discussed in subsequent papers (Skejo et al., 2021; Raval et al., 2022; Bremer et al., 2023). Importantly, Garg and Martin assumed that primitive kinetochores were already present in the syncytial ancestor to allow duplicated chromosomes to be pushed apart by microtubules (Garg and Martin, 2016). Below, I challenge this assumption.

## Two different types of kinetochores exist in eukaryotes

Chromosome segregation in all present-day eukaryotes studied thus far relies on spindle microtubules (McIntosh et al., 2010). Indeed, tubulins are conserved in all known eukaryotes (Findeisen et al., 2014). In contrast, despite the essentiality of kinetochores for the survival of all present-day eukaryotes, at least two kinetochore systems with distinct compositions exist – the canonical kinetochore proteins present in almost all eukaryotes (Meraldi et al., 2006; Drinnenberg and Akiyoshi, 2017; van Hooff et al., 2017) and the unconventional kinetochore proteins found only in kinetoplastids (Akiyoshi, 2016). Both types of kinetochores have more than 20 components, and how such complex molecular machines evolved is an interesting topic of discussion. Canonical kinetochore proteins include CENP-A, a centromere-specific histone H3 variant that specifies the position of kinetochore assembly in many eukaryotes (Earnshaw and Rothfield, 1985), and the microtubule-binding Ndc80 complex (Cheeseman et al., 2006; Wei et al., 2007). Bioinformatics analysis has shown that canonical kinetochore proteins are of mosaic origin, meaning that they were repurposed from various other biological processes, such as intraflagellar transport (Tromer et al., 2019). In contrast, it has been proposed that kinetoplastid kinetochore (KKT) proteins evolved by repurposing meiotic chromosome synapsis and homologous recombination machineries (Tromer et al., 2021). So far, no eukaryote is known to have both types of kinetochore systems, demonstrating an evolutionary dichotomy in eukaryotic chromosome segregation machines.

**Fig. 2.**
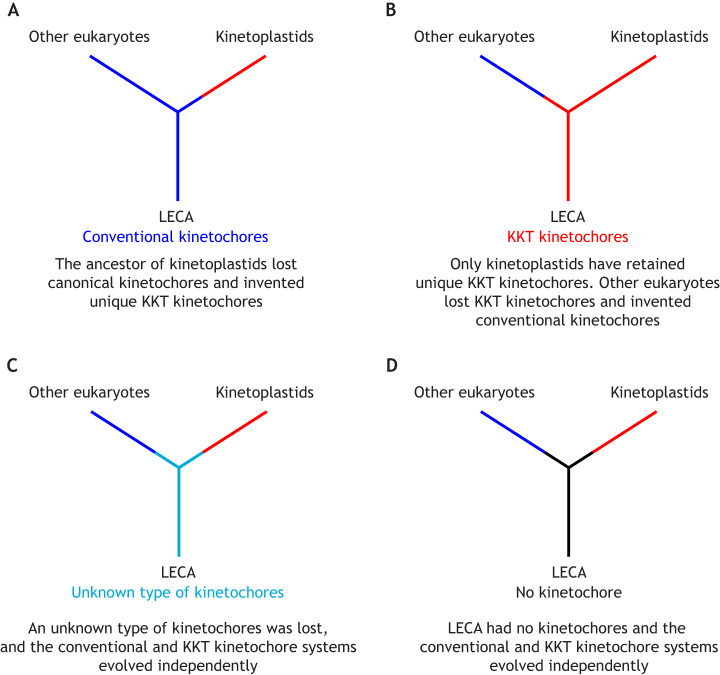
**Four scenarios for the evolution of two kinetochore systems under the earliest-branching kinetoplastids hypothesis.** (A) If the LECA had conventional kinetochores, the ancestor of kinetoplastids abandoned the conventional kinetochores and replaced them with KKT-based kinetochores. (B) If the LECA had KKT-based kinetochores, the ancestor of non-kinetoplastids abandoned the KKT kinetochores and replaced them with conventional kinetochores. (C) If the LECA had an unknown type of kinetochore, such kinetochores were lost and the two present-day kinetochore systems evolved independently. (D) If the LECA did not have a kinetochore, the two kinetochore systems evolved independently after kinetoplastids split from the rest of eukaryotes. This scenario does not require replacement of kinetochore systems. Conventional, KKT-based, and unknown kinetochore types are shown in blue, red and cyan, respectively. This figure was adapted and modified from Akiyoshi (2016), where it was published under a CC-BY 4.0 license.

Whether the ancestor of kinetoplastids had a canonical kinetochore system remains unknown. This is because we still do not know the position of the root of the eukaryotic tree of life, which remains a highly controversial topic with various competing ideas proposed over the years (Box 2). Based on the wide distribution of canonical kinetochore proteins among eukaryotes, the LECA has been deduced to have possessed canonical kinetochores (Tromer et al., 2019). However, this deduction relies on the assumption that kinetoplastids are not one of the earliest-branching eukaryotes, a crucial point that cannot be ignored. In fact, it has been proposed that kinetoplastids (Euglenozoa) are among the earliest-branching eukaryotes (Cavalier-Smith, 2010a; Akiyoshi and Gull, 2014). If this is the case, there are at least four possibilities for what kind of kinetochores were present in the LECA (Fig. 2). The first possibility is that the ancestor of kinetoplastids had canonical kinetochores, which were eventually lost and replaced by the KKT system (Fig. 2A). I deem this possibility unlikely because it would require the kinetoplastid ancestor to have abandoned the canonical kinetochore (an excellent chromosome segregation machine essential for its survival) and re-invented a different machinery. However, I certainly cannot exclude this possibility, because striking molecular transitions have been found to occur for many processes; for example, the loss of canonical nucleosomal DNA packaging in dinoflagellates (Gornik et al., 2012). The second possibility is that kinetoplastids never had canonical kinetochores because the LECA possessed KKT kinetochores (Fig. 2B). Again, this would involve loss of a robustly functional system (the KKT kinetochore) and invention of the canonical kinetochore system in the ancestor of non-kinetoplastid eukaryotes. The third possibility is that the LECA possessed a hitherto unknown type of kinetochore, which was lost at some point in eukaryotic evolution (Fig. 2C). The fourth possibility is that kinetoplastids never had canonical kinetochores because the LECA had no kinetochore, and that the two distinct kinetochore systems evolved independently (Fig. 2D). It is important to note that the fourth scenario would nicely explain the evolutionary dichotomy of kinetochores in eukaryotes. However, if this scenario is the case, how then did the LECA segregate its chromosomes? As mentioned above, in prokaryotes that lack kinetochores, an entropy-based mechanism has been proposed as a major driving force of genome segregation (Jun and Mulder, 2006; Jun and Wright, 2010; Jun, 2015; Polson and Zhu, 2021). If prokaryotes can segregate their chromosomes without an active segregation machinery, it is conceivable that ancestral eukaryotes segregated their chromosomes without a sophisticated kinetochore machinery. Such a scenario would imply the absence of mitosis at this stage.

Box 2. Where is the root of the eukaryotic tree of life? All known eukaryotes are thought to be descended from a common ancestor (Richards et al., 2024; Vosseberg et al., 2024). However, it remains unclear which organisms diverged at the earliest point in eukaryotic evolution (Embley and Martin, 2006; Walker et al., 2011; Burki et al., 2020). There is no consensus on the position of the root of the eukaryotic tree of life, with many competing hypotheses proposed over the years (Stechmann and Cavalier-Smith, 2003; Richards and Cavalier-Smith, 2005; Rogozin et al., 2009; Cavalier-Smith, 2010a; Derelle and Lang, 2012; Katz et al., 2012; He et al., 2014; Derelle et al., 2015; Cerón-Romero et al., 2022; Al Jewari and Baldauf, 2023; Torruella et al., 2025; Williamson et al., 2025). This lack of consensus could be interpreted as evidence that even modern phylogenetic approaches cannot resolve the eukaryotic root, simply due to the extreme evolutionary distance between eukaryotes and prokaryotes. To determine root organisms within a given group, standard sequence-based phylogenetic approaches require comparison to an outgroup organism that does not belong to the group but is reasonably closely related to it. Thus, to determine the eukaryotic root using modern phylogenetics, the outgroup organism must be a prokaryote. However, all known prokaryotes (even the Asgard archaea identified so far) are extremely divergent from all known eukaryotes, which could explain the origin of competing hypotheses. Some studies have used bacterial-origin proteins to infer the root position, reaching different conclusions (Derelle and Lang, 2012; He et al., 2014; Derelle et al., 2015; Torruella et al., 2025; Williamson et al., 2025). It is noteworthy that neither kinetoplastids nor diplonemids were included in recent analyses (Torruella et al., 2025; Williamson et al., 2025), which used *Euglena* as the representative of Euglenozoa.

Without a proper outgroup, many recent phylogenetic eukaryotic trees classify organisms into groups based on similarities among them and cannot be used to determine root organisms. In other words, the fact that kinetoplastids do not appear as the root organisms within Euglenozoa in such trees (e.g. Keeling and Burki, 2019) does not exclude the possibility that kinetoplastids, or kinetoplastids-diplonemids (glycomonads), are the root organisms for all eukaryotes.

## Archaea have machineries for cell fusion and genetic exchange

Archaea do not have kinetochores. By contrast, archaea do exhibit evidence of cell fusion and genetic exchange (Gross and Bhattacharya, 2010; White, 2011; Wagner et al., 2017). In fact, archaeal homologs are present for HAP2, a membrane fusion protein required for gamete cell fusion (Moi et al., 2022), as well as Spo11, its cofactor TOPOVIBL (also known as Rec102) and Dmc1, all of which are involved in meiotic recombination (Sandler et al., 1996; Bergerat et al., 1997; Keeney et al., 1997; Robert et al., 2016; Vrielynck et al., 2016; Bernstein and Bernstein, 2017). Together with the widespread distribution of these proteins in present-day eukaryotes (Ramesh et al., 2005; Malik et al., 2007a,b; Speijer et al., 2015; Brinkmeier et al., 2022; Allen and Maxwell, 2024), these findings strongly support the possibility that the LECA was capable of cell fusion and genetic exchange.

## Hypothesis – ancestral eukaryotes sexually proliferated without kinetochores or mitosis

It is widely accepted that eukaryotes evolved from an archaeon that incorporated a bacterial symbiont (Embley and Martin, 2006; Williams et al., 2013). It is therefore conceivable that ancestral eukaryotes had access to machineries that are present in archaea (bearing in mind an important caveat that the archaeon that underwent eukaryogenesis might be fundamentally different from any present-day archaea). Based on the evidence discussed above, I propose that ancestral eukaryotes were capable of cell fusion and genetic exchange but did not have a kinetochore-dependent genome segregation mechanism, which would constitute a primitive meiosis-like state in the absence of mitosis. In this state, genetically exchanged chromosomes would segregate spontaneously via an entropy-driven mechanism, likely within the confinement of the nucleus (i.e. closed mitosis), which might correspond to the cytoplasm of an archaeal host (Baum and Baum, 2014). Alternatively, the nucleus might not yet have evolved at this stage. Although it is unknown whether the chromosomes were circular or linear, an entropy-driven segregation mechanism could work for both types of chromosomes (Jun and Mulder, 2006). I envisage existence of a prokaryote-like simple genome at this stage and do not assume the presence of the sophisticated meiotic mechanisms that operate in present-day organisms, such as mono-orientation of sister kinetochores.

My proposal − that some aspects of meiosis evolved prior to mitosis − is much the same as that introduced by Garg and Martin (2016), with a few key differences. Garg and Martin proposed a syncytial eukaryote common ancestor with multiple nuclei that exchanged genetic material. In my view, such a cell does not need to be syncytial if cell fusion machinery (HAP2) is present, which is more consistent with the idea that the LECA was likely a population of cells rather than a single cell (O'Malley et al., 2019). Most importantly, I do not assume the presence of a kinetochore-based chromosome segregation mechanism, a key basis for my hypothesis. However, I share their idea that chromosome segregation continued until only one genome copy per nucleus remained because “given the tools and the energy, chromosome segregation does not know when to stop” (Garg and Martin, 2016), which can be viewed as the origin of reductional segregation (segregation resulting in a reduction in chromosome number, a defining feature of meiosis in present-day eukaryotes). Although this primitive-meiosis-first hypothesis might sound provocative, it can, in my view, rationally explain several phenomena, as I discuss below.

## Implication 1 – mitosis evolved as a streamlined form of meiosis

A serious issue associated with the dogma that meiosis evolved from mitosis is that it is difficult to explain the molecular events that enabled such a transition. In fact, Maynard Smith and Hamilton regarded the origins of meiosis as one of the most difficult evolutionary problems (Maynard Smith, 1978; Hamilton, 1996). Although meiosis and mitosis share many similarities, the former is much more complicated than the latter. The transition from mitosis to meiosis would therefore have required multiple steps. In contrast, under the hypothesis that key features of meiosis were already present prior to the origin of mitosis, we would only need to explain the evolution of present-day meiosis (and mitosis) from the primitive meiosis-like state. I speculate that once the primitive meiosis-like mechanism evolved, an active chromosome segregation machinery (kinetochores) subsequently evolved to allow accurate segregation of more complex genomes. In present-day eukaryotes, meiosis I involves chromosome pairing, linkage of homologous chromosomes by chiasmata, mono-orientation of kinetochores and protection of centromeric cohesin to allow segregation of homologous chromosomes (Petronczki et al., 2003). All of these phenomena should be feasible through additions and modifications to existing mechanisms. In this context, mitosis can be viewed as a streamlined form of meiosis, providing yet another reason why it would intuitively make more sense to think that mitosis evolved from the meiosis-like state.

## Implication 2 – mitotic chromosome arm cohesin is a relic of meiotic synapsis

Cohesin, a eukaryote-specific invention (van Hooff et al., 2024 preprint), mediates cohesion between duplicated chromosomes in eukaryotes (Guacci et al., 1997; Michaelis et al., 1997). During meiosis, cohesin is also important for promoting chromosome synapsis, genetic exchange and mono-orientation of sister chromatids (Klein et al., 1999; Watanabe and Nurse, 1999). When homologous chromosomes separate during meiosis I, centromeric cohesin is protected from degradation through recruitment of the protein phosphatase PP2A to maintain cohesion of sister chromatids at centromeres, thereby allowing their segregation during meiosis II (Kitajima et al., 2006; Riedel et al., 2006).

In mitotically dividing cells, centromeric cohesin is sufficient to provide linkage between sister chromatids (Losada et al., 1998; Sumara et al., 2000). However, cohesin complexes also exist on chromosome arms (Lengronne et al., 2004), and the evolutionary origin of arm cohesin remains unclear. Under the primitive-meiosis-first hypothesis, the arm cohesin in mitotic cells can be interpreted as a relic of the chromosome-wide cohesin used to promote recombination during meiosis. In fact, during mitosis in human cells, the majority of cohesin on chromosome arms is removed, whereas centromeric cohesin is protected by PP2A, a mechanism similar to that used in meiosis I (Hauf et al., 2005; McGuinness et al., 2005; Shintomi and Hirano, 2009; Nishiyama et al., 2013). Finally, it is noteworthy that DNA damage in mitotically dividing cells can cause genome-wide accumulation of cohesin complexes (Ström et al., 2007; Unal et al., 2007), which could also be viewed as a relic of meiotic synapsis.

## Implication 3 – PLK1 has enhanced roles in meiosis compared to mitosis

The mitotic kinase polo-like kinase 1 (PLK1) performs various functions, including regulation of homologous recombination, synaptonemal complexes, kinetochores and cytokinesis (Archambault and Glover, 2009; Zitouni et al., 2014; Cahoon and Hawley, 2016). Interestingly, in some organisms, including fission yeast and budding yeast, PLK1 plays crucial roles in chromosome segregation in meiosis I but not in mitosis (Lee and Amon, 2003; Kim et al., 2015). These findings could be better explained if PLK1 had an important role in the primitive meiosis-like mechanism in ancestral eukaryotes and continued to be important for chromosome segregation in meiosis but became less important for mitotic chromosome segregation. Localization of PLK1 to kinetochores during mitosis in animals could represent a more recent adaptation to achieve accurate chromosome segregation of large genomes (Elowe et al., 2007; McKinley and Cheeseman, 2014). Future studies of PLKs in divergent eukaryotes could test this idea. For example, *Trypanosoma brucei* PLK does not even localize within the nucleus during mitosis (Kurasawa et al., 2020). It will be interesting to examine whether PLK plays a role in chromosome segregation during meiosis in this organism.

## Implication 4 – the root of the eukaryotic tree of life lies between kinetoplastids, or kinetoplastids-diplonemids, and the rest of eukaryotic life

According to my hypothesis that the LECA did not have a kinetochore, the presence of unique kinetochore proteins in kinetoplastids does not necessitate a replacement of the canonical kinetochore system with a unique one (which I deem unlikely). Instead, I have argued that the two kinetochore systems found in present-day eukaryotes evolved independently after kinetoplastids branched off from the rest of the eukaryotes (Fig. 2D). This could also better explain the lack of any overlap between the two kinetochore systems in any known eukaryotic lineages.

Determining the position of the root of the eukaryotic tree of life remains an unresolved problem (Burki et al., 2020) (Box 2). A corollary of my hypothesis is that the presence of the unique kinetochore system in kinetoplastids supports the idea that kinetoplastids are one of the earliest-branching eukaryotes. Kinetoplastids (Euglenozoa) have many unique or highly divergent molecular features, including in mRNA processing and splicing (Clayton, 2002; Castañeda Londoño et al., 2021), mitochondrial *c*-type cytochrome biogenesis (Allen et al., 2008; Belbelazi et al., 2021), mitochondrial DNA organization (Borst, 2016; Schneider and Ochsenreiter, 2018), glycosomes (Gabaldón et al., 2016), nuclear lamina and the nuclear pore complex (Koreny and Field, 2016; Butterfield et al., 2024). These features are typically interpreted as derived states, because many of them were originally discovered in parasites, which often evolve unique mechanisms to maximize their survival. I argue that these unique features, many of which are conserved in free-living kinetoplastids, actually support the possibility that kinetoplastids are early branching.

Kinetoplastids belong to Euglenozoa, which also includes diplonemids, euglenids and symbiontids (Kostygov et al., 2021). Among Euglenozoa, kinetoplastids and diplonemids are grouped together in a clade called glycomonads (Cavalier-Smith, 2016). It is noteworthy that diplonemids might possess another hitherto unknown kinetochore system, whereas euglenids have canonical kinetochores (Butenko et al., 2020; Benz et al., 2024; Akiyoshi et al., 2025 preprint). Identifying kinetochore components in diplonemids will help shed further light on the position of the root of eukaryotic tree of life as well as evolution of chromosome segregation machines in eukaryotes.

## Conclusions

In this Hypothesis article, I have discussed the mechanisms of chromosome segregation in all domains of life. By speculating on the mechanism used in ancestral eukaryotes, I have proposed that the evolution of (some aspects of) meiosis preceded that of mitosis, an idea which was initially proposed by Garg and Martin based on an evolutionary genetics theory (Garg and Martin, 2016). Importantly, I reached this idea independently through discussion of a eukaryote-specific feature of chromosome segregation, the kinetochore. I appreciate that proving this hypothesis is almost impossible, partly because eukaryogenesis can still not be reproduced in the laboratory. Furthermore, the huge gap between any known present-day eukaryotes and prokaryotes makes the study of eukaryogenesis by means of traditional phylogenetic approaches extremely challenging and prone to artifacts. In the future, discovery of more eukaryote-like archaea or more prokaryote-like eukaryotes could shed further light on this key evolutionary transition.
